# A Flexible and Wearable Photodetector Enabling Ultra‐Broadband Imaging from Ultraviolet to Millimeter‐Wave Regimes

**DOI:** 10.1002/advs.202401631

**Published:** 2024-04-24

**Authors:** Shaojing Liu, Ximiao Wang, Ningsheng Xu, Runli Li, Hai Ou, Shangdong Li, Yongsheng Zhu, Yanlin Ke, Runze Zhan, Huanjun Chen, Shaozhi Deng

**Affiliations:** ^1^ State Key Laboratory of Optoelectronic Materials and Technologies Guangdong Province Key Laboratory of Display Material and Technology School of Electronics and Information Technology Sun Yat‐sen University Guangzhou 510275 China

**Keywords:** broadband, flexible, graphene, photodetectors, terahertz imaging

## Abstract

Flexible and miniaturized photodetectors, offering a fast response across the ultraviolet (UV) to millimeter (MM) wave spectrum, are crucial for applications like healthcare monitoring and wearable optoelectronics. Despite their potential, developing such photodetectors faces challenges due to the lack of suitable materials and operational mechanisms. Here, the study proposes a flexible photodetector composed of a monolayer graphene connected by two distinct metal electrodes. Through the photothermoelectric effect, these asymmetric electrodes induce electron flow within the graphene channel upon electromagnetic wave illumination, resulting in a compact device with ultra‐broadband and rapid photoresponse. The devices, with footprints ranging from 3 × 20 µm^2^ to 50 × 20 µm^2^, operate across a spectrum from 325 nm (UV) to 1.19 mm (MM) wave. They demonstrate a responsivity (*R*_V_) of up to 396.4 ± 5.1 mV W^−1^, a noise‐equivalent power (NEP) of 8.6 ± 0.1 nW Hz^−^^0.5^, and a response time as small as 0.8 ± 0.1 ms. This device facilitates direct imaging of shielded objects and material differentiation under simulated human body‐wearing conditions. The straightforward device architecture, aligned with its ultra‐broadband operational frequency range, is anticipated to hold significant implications for the development of miniaturized, wearable, and portable photodetectors.

## Graphical abstract

A flexible and wearable graphene photodetector, leveraging the photothermoelectric effect, enables ultra‐broadband and rapid photoresponse spanning from ultraviolet to millimeter waves at room temperature. Importantly, the device eliminates the need for antennas in terahertz and millimeter wave detection, simplifying the overall architecture. Under simulated human body‐wearing conditions, the device facilitates wide‐spectrum imaging of shielded objects and material differentiation.

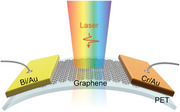

## 1 Introduction

Flexible, compact, and high‐speed photodetectors with a broad UV to MM wave response offer substantial potential for applications in healthcare monitoring, electronic skin, environmental sensing, advanced communication systems, remote detection and wearable biomedical tools.^[^ ^1^ ^−^ ^6^ ^]^ However, realizing this technology poses a significant challenge due to the lack of suitable materials and detection mechanisms. First, most existing photodetectors rely on rigid and fragile semiconductors like Si, Ge, InGaAs, HgCdTe, PbSe, etc., making them unsuitable for integration into mechanically flexible substrates.^[^ ^7^ ^−^ ^10^ ^]^ Second, these traditional semiconductors exhibit narrow detection bands limited by the energy gaps associated with interband, intraband, or inter‐subband optical transitions, hindering broadband operation. Third, current terahertz (THz) detectors in the market, including Golay cells, thermopiles, and bolometers, along with proposed THz detectors, require intricate integration with antennas, posing challenges for miniaturization.^[^ ^11^ ^−^ ^15^ ^]^ Overcoming these obstacles is essential for the development of ultra‐broadband and compact room‐temperature flexible photodetectors.

Due to their unique optoelectronic properties and mechanical stretchability, van der Waals 2D materials have ignited significant interest as a potential candidate for developing flexible photodetectors (Table S1, Supporting Information). For instance, previous investigations have illustrated the fabrication of two‐terminal‐geometry devices on flexible substrates utilizing 2D semiconductors like transition metal dichalcogenides and black phosphorous. These devices, designed for lightweight and shape‐conforming flexible photodetectors, show impressive responsivity, specific detectivity (*D* ^*^), and response times of up to 1.28 × 10^3^ A W^−1^, 3.02 × 10^11^ Jones, and millisecond, respectively. However, the finite band gaps of 2D semiconductors limit the spectral response of these flexible photodetectors to the UV to near‐infrared (NIR) region.^[^ ^16^ ^−^ ^22^ ^]^ In contrast, 2D semimetals (e.g., graphene, WTe_2_, PtTe_2_, PdTe_2_, NiTe_2_, and T_d_‐MoTe_2_), characterized by gapless band structures, have recently garnered interest for the development of photodetectors with a broadband response (Table S1, Supporting Information). Notably, type‐II semimetals like WTe_2_,^[^ ^23^ ^]^ Cd_3_As_2_,^[^ ^24^ ^]^ and T_d_‐MoTe_2_,^[^ ^25^ ^]^ featuring heavily tilted linear electronic dispersions, exhibit fast photodetection across broad spectral ranges from UV to THz regimes, driven by photoexcited hot carriers navigating the tilted band structure. Nevertheless, most reported 2D semimetal‐based photodetectors are confined to rigid substrates. Even on flexible substrates,^[^ ^6^, ^26^, ^27^ ^]^ inefficient hot carrier generation necessitates additional antennas or large channel lengths for long‐wavelength photodetection. This results in a large device footprint, slow response speed, and intricate fabrication, constraining their use in miniaturized and flexible devices for detection and imaging.

In this study, we introduce a flexible and wearable photodetector utilizing a semimetal, monolayer graphene, connected by two dissimilar metal electrodes. The photodetector operates in a self‐powered mode at room temperature, exploiting the hot‐carrier photothermoelectric (PTE) mechanism. In this context, photoexcited carriers generate a net current flow propelled by the carrier temperature gradient induced by the two dissimilar contacts. With a compact size of 3 µm × 20 µm, it covers an ultra‐broadband spectrum from UV (325 nm) to MM waves (1.19 mm). The device demonstrates an *R*_V_ of 0.3 ± 0.1 mV W^−1^ (at 325 nm), 1.5 ± 0.2 mV W^−1^ (at 532 nm), 1.04 ± 0.03 mV W^−1^ (at 785 nm), 6.7 ± 0.3 mV W^−1^ (at 10.5 µm), and 396.4 ± 5.1 mV W^−1^ (at 1.11 mm), with the corresponding NEP of 13.7 ± 4.8 µW Hz^−^^0.5^, 2.2 ± 0.2 µW Hz^−^^0.5^, 3.3 ± 0.1 µW Hz^−^^0.5^, 505.5 ± 23.9 nW Hz^−^^0.5^, and 8.6 ± 0.1 nW Hz^−^^0.5^, respectively. Additionally, it exhibits response times of 0.8 ± 0.1 ms (at 325 nm), 2.0 ± 0.2 ms (at 532 nm), 1.4 ± 0.1 ms (at 785 nm), 61.8 ± 1.5 ms (at 10.5 µm), and 697 ± 13 ms (at 119 µm). Importantly, the device eliminates the need for antennas in THz and MM waves detection, simplifying the overall architecture. When conformally attached to simulated curved surfaces of the human body, the flexible photodetector enables wide‐spectrum imaging of shielded objects and various materials, showcasing its potential for ultra‐broadband and smart optoelectronic devices.

## 2 Fabrication of the Flexible Photodetectors

**Figure 1:**
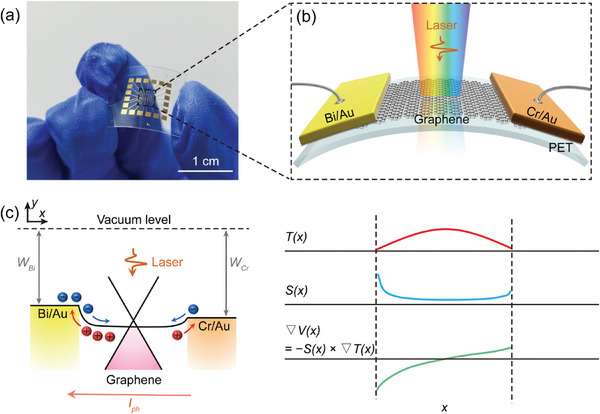
Flexible graphene photodetector and its operation principle. a) Photograph of the flexible graphene photodetector. b) Schematic showing the flexible photodetector, composed of asymmetric metal electrodes (Bi/Au and Cr/Au) connected with a monolayer graphene as the channel. c) Left: band diagram of the photodetector. Right: profiles across the photodetector of electron temperature *T*(*x*), Seebeck coefficient *S*(*x*), and potential gradient ∇V(*x*).

The flexible and transparent photodetector was fabricated by transferring monolayer graphene onto polyethylene terephthalate (PET) substrate and possesses a simple two‐terminal architecture (Experimental Section and Figure S1, Supporting Information). As illustrated in **Figure** **1**a,b, the photodetector incorporates metals Bi (10 nm in thickness) and Cr (10 nm in thickness) to establish contacts with the monolayer graphene film at the two terminals. A 100‐nm Au layer was deposited onto the two metals for subsequent wire bonding. It is noteworthy that the choice of Bi metal as one of the electrodes, distinguished by a considerable disparity in work function compared to Cr metal (*W*_Cr_ − *W*_Bi_ = 0.28 eV) and characterized by a low melting point,^[^ ^28^ ^−^ ^30^ ^]^ not only facilitates the photodetection via PTE but also ensures minimal damage to the device during electrode deposition.

Figure 1c elucidates the operation principle of the Bi/Au–graphene–Cr/Au photodetector. Specifically, incident light elevates the temperature of electrons within the monolayer graphene, while the asymmetrical electrode contacts serve as heat sinks, resulting in a non‐uniform temperature distribution, *T*(*x*), along the channel. Owing to the different work function of the Bi and Cr, the metal‐induced doping of graphene varies at the two terminals, giving rise to a nonuniform local Fermi energy, *E_f_*, across the channel region. This will result in a position‐dependent Seebeck coefficients (*S*), which can be calculated as:^[^ ^31^, ^32^ ^]^

(1) $$S=-\frac{\pi^{2}{k_{B}}^{2}T}{3e}{\left.\left(\frac{d\ln\sigma }{dE}\right)\right|}_{E=E_{f}}$$

where *k_B_* is Boltzmann constant, *T* is absolute temperature, *e* is the elementary charge and *σ* is material's electrical conductivity. It is noted that the *E_f_* in Equation (1) is defined according to the vacuum level, i.e., the *E*_*f*_ is negative. The temperature gradient ∇*T*(*x*) gives rise to a local potential ∇*V*(*x*) expressed as ∇*V*(*x*) = −*S*(*x*)·∇*T*(*x*) (Figure 1c, right panel), which facilitates the diffusion of hot carriers. Specifically, at the graphene−Bi contact, ∇*V*(*x*) is negative, resulting in a negative photocurrent, while the photocurrent is positive at the graphene−Cr contact. Due to the differences in work functions among the metals and graphene (*W*_Gr_ > *W*_Cr_ > *W*_Bi_),^[^ ^28^, ^29^, ^33^ ^]^ the ∇*V*(*x*) at the graphene−Bi contact is larger than that at the graphene−Cr contact, leading to a higher photocurrent at the graphene−Bi contact (Figure 1c, left panel). Therefore, under uniform illumination of the device, a net photocurrent flowing toward the negative direction will be observed. This will result in a net photovoltage, *V*_ph_, between the two device terminals, which can be calculated by integrating ∇*V(x)* along the channel length *L*_c_,^[^ ^34^ ^]^

(2) $$V_{\mathrm{ph}}=\int_{0}^{\mathrm{Lc}}\nabla V\left(x\right)\mathrm{dx}=-\int_{0}^{\mathrm{Lc}}S\left(x\right)\nabla T\left(x\right)\mathrm{dx}$$

## 3 Characterizations of Flexible Photodetectors

**Figure 2:**
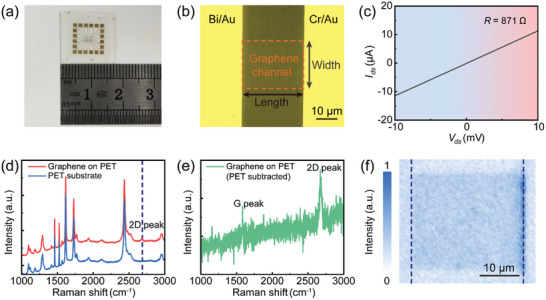
Characterization of the flexible graphene photodetector. a) Photograph of the flexible graphene photodetector array. b) Optical microscope image of a typical flexible graphene photodetector with channel length of 30 µm and width of 20 µm. The graphene channel, represented in the orange dashed box, contacts with Bi/Au electrode (left) and Cr/Au electrode (right), respectively. c) Dependence of current on applied voltage of the flexible graphene photodetector. d) Raman spectra of a monolayer graphene on PET substrate (red curve) and clean PET substrate (blue curve). The 2D peak of graphene is indicated by the black dashed line. e) Raman spectrum of monolayer graphene on a PET substrate, obtained by subtracting the PET substrate signal from the total signals represented by red curve shown in Figure 2d. f) 2D Raman mapping of the intensity at the 2D peak within the graphene channel. The black dashed lines show the boundaries between metal electrodes and graphene.

An array of flexible photodetectors with fixed channel width at 20 µm while varying channel lengths ranging from 3 to 50 µm was fabricated on a 1.7 × 1.7 cm^2^ PET substrate, as depicted in **Figure** **2**a. Figure 2b shows the optical microscope image of a typical photodetector with a 30‐µm channel length, chosen for subsequent photodetection (additional devices are presented in Figure S2a, Supporting Information). The current–voltage (*I*–*V*) characteristic of the photodetector without external illumination, as illustrated in Figure 2c, exhibits a linear relationship between current and bias voltage, suggesting the good Ohmic contacts between graphene and two electrodes. The calculated resistance from the *I*–*V* characteristic is 871 Ω at *L*_c_ = 30 µm, and increasing from 131 ± 8 Ω to 1014 ± 16 Ω with the extension of the channel length from 3 to 50 µm (Figure S2b, Supporting Information). This variation highlights the excellent conductivity of graphene, which is significant for the observable photoresponse as discussed below.

Raman measurements were conducted to evaluate the quality and uniformity of the monolayer graphene. Because there is a polymer mask coated onto the monolayer graphene film obtained commercially (Jiangsu XFNANO Materials Tech. Co., Ltd.), it is difficult to directly measure the Raman spectrum from the monolayer graphene. We therefore transferred the monolayer graphene onto a Si/SiO_2_ substrate and conducted Raman characterization after removing the polymer layer. The results indicate two distinct characteristic peaks, which are the 2D peak at ≈2700 cm^−1^ and the G peak at ≈1580 cm^−1^, with a peak intensity ratio *I*_2D_/*I*_G_ approximately equal to 1.86 (Figure S3, Supporting Information). Additionally, a relatively weak intensity of the D peak at ≈1350 cm^−1^ is noted. These results indicate the high crystallinity and monolayer nature of the graphene. We also conducted Raman spectroscopic measurement on the graphene transferred to the PET substrate. As depicted in Figure 2e, upon subtracting the background Raman spectrum of the PET substrate (represented by the blue curve in Figure 2d) from that of graphene on the PET substrate (illustrated by the red curve in Figure 2d), the two distinct characteristic peaks associated with monolayer graphene—the 2D peak and G peak—are observable at ≈2700 and 1580 cm^−1^, respectively. Furthermore, a peak intensity ratio of *I*_2D_/*I*_G_ approximately equal to 1.32 is observed, suggesting that the monolayer graphene maintains its high crystallinity even after being transferred to the PET substrate. Moreover, a 2D Raman mapping of the intensity at the 2D peak, presented in Figure 2f, reveals a smooth distribution with minimal fluctuation, providing further confirmation of the uniformity and integrity of the graphene channel. This result suggests that the graphene remains intact throughout the transfer and micro‐nanofabrication processes.

## 4 Ultra‐Broadband and Fast Photoresponse of the Flexible Photodetectors

**Figure 3:**
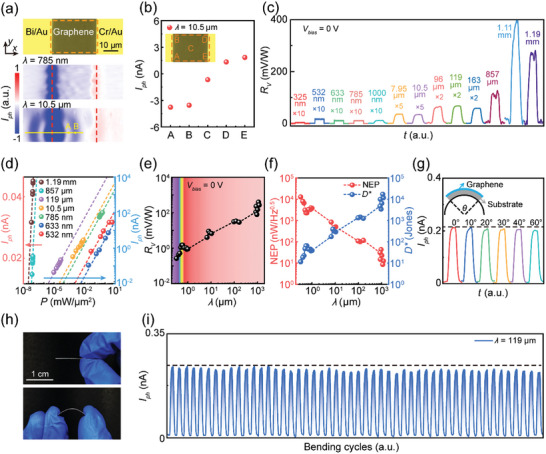
Photodetection performances of the flexible graphene device. a) Optical microscope image (upper panel) and scanning photocurrent microscopy images of the flexible graphene photodetector. The photocurrent images were collected using illumination wavelengths of 785 nm (middle panel) and 10.5 µm (lower panel) without any bias. The orange dashed box and red lines mark the graphene channel and metal–graphene contacts, respectively. Point A and point B represent the centers of dark and bright fringes, respectively. b) Photocurrent (*I_ph_*) of the flexible graphene photodetector recorded at five typical positions in the graphene channel. The illumination wavelength is 10.5 µm. c) Time‐resolved photocurrent responses in a broadband spectrum. For a better comparison, the *R*_V_ associated with several illumination wavelengths are multiplied by specific factors. d) Dependence of the photocurrent on the illumination power at various wavelengths. Dots: experimental results. Lines: linear fittings. The data associated with 857‐µm and 1.19‐mm illumination correspond to the left *y*‐axis, while those associated with illumination of other wavelengths correspond to the right *y*‐axis. e) Wavelength‐dependent *R*_V_ of the flexible graphene photodetector. f) NEP (red dots) and *D* ^*^ (blue dots) of the flexible graphene photodetector as a function of illumination wavelength. g) Time‐resolved photocurrent response of the photodetector upon different bending angles. Inset: schematic showing the definition of *θ*. h) Photographs of the flexible graphene photodetector at its free state and bending state. i) Time‐resolved photocurrent of the photodetector during 50 repeated bending cycles. The bending angle is 60°. The illumination wavelength in (g) and (i) is 119 µm (2.52 THz).

The photoresponse of the flexible photodetector was assessed by measuring its photocurrent generation under illumination with various wavelengths (Figure 1b). Notably, discernible photocurrent signals were recorded across a range of excitation wavelengths spanning from UV (325 nm) to the MM wave (1.19 mm) region, all while maintaining a zero bias between the two electrodes of the photodetector (**Figure** **3**c). To the best of our knowledge, this represents the broadest range reported for flexible photodetectors based on 2D materials (Table S1, Supporting Information). This self‐powered and ultra‐broadband operational mode strongly signifies that the photocurrent generation mechanism in the flexible device is the PTE effect. In this mechanism, the photocurrent is induced by the diffusion of electrons, driven by the electron temperature gradient, without the need for any bias voltage. To further validate this mechanism, the distribution of photocurrent within the device, covering both the graphene channel and the overlap regions of metal–graphene contacts, was visualized using scanning photocurrent microscopy (SPCM) (refer to Experimental Section for detailed information on the SPCM measurements). To prevent heating damage to graphene caused by high laser power, the photocurrent scanning was conducted at a power of 1 mW.^[^ ^35^ ^]^ As illustrated in Figure 3a, under illumination with a 785‐nm laser, a negative photocurrent is generated at the graphene–Bi contact (left terminal), while the photocurrent polarity reverses at the graphene–Cr contact (right contact). This distinct behavior persists when utilizing a 10.5‐µm laser as the excitation source, as demonstrated in the lower panel of Figure 3a. Noteworthy is the observation that within the channel region, the photocurrent undergoes a transition from negative to positive as the laser spot is scanned from the Bi electrode to the Cr electrode. The photocurrent disappears entirely when the laser spot is positioned at the midpoint of the channel (refer to Figure 3a,b). Furthermore, for both the 785‐nm and 10.5‐µm illuminations, the photocurrent near the graphene–Bi contact is larger than that at the graphene–Cr contact (Figure 3a). To illustrate this behavior more clearly, the position‐dependent photocurrent profiles under the two illuminations were measured along the midline of the detector channel (lower panel of Figure S4d,e, Supporting Information). In both cases, the results exhibit a significant difference in photocurrent intensity between the graphene–Bi contact and the graphene–Cr contact, resulting in a pronounced net photocurrent. All these features are consistent with the analysis shown in Figure 1c, and therefore collectively confirm that the operating mode of the device is the PTE. In the subsequent discussion on photocurrent, the laser spot will be focused in the vicinity of the graphene–Bi contact, and fine adjustment will be made to optimize the photocurrent under a specific illumination wavelength.

One interesting phenomenon is that when the wavelength is 10.5 µm (Figure 3a, lower panel), the photocurrent in the channel region does not change monotonically along the *x*‐axis direction. We believe that such a phenomenon is due to the diffraction effect. Specifically, at *λ* = 10.5 µm, the separation between dark (point A) and bright fringes (point B) along the *x*‐axis is ≈5 µm, precisely half of the incident wavelength, suggesting a diffraction of the laser by the edge of the contact. However, for the 785‐nm illumination, half of the wavelength is 392.5 nm, which presents challenges in resolving it distinctly in imaging due to its small size. Such a size is beyond the resolution of our photocurrent imaging system. Therefore, the non‐monotonic photocurrent distribution along the channel does not appear.

As a comparison, we also fabricated a detector using the same Bi electrodes and studied its photoresponse by measuring the photocurrent generated within the excitation wavelength range spanning from 325 nm to 1.19 mm. Evident photocurrent signals were recorded across a range of excitation wavelengths spanning from UV (325 nm) to mid‐infrared (MIR) (10.5 µm) regions (Figure S4a, Supporting Information). However, negligible photoresponse was observed for the THz and MM wave illuminations. This can be attributed to the fact that the laser spots of the THz to MM wave illuminations are larger than the channel region, resulting in both of the two contacts being covered by the lasers. Consequently, due to the equal barrier heights but with opposite polarity at the two graphene–Bi contacts, the photocurrent generated at these two contacts will cancel each other out. To investigate this mechanism more deeply, the distribution of photocurrent across the detector channel and the overlap regions of graphene–Bi contacts were visualized using SPCM (Figure S4b,c, Supporting Information). Under illumination with a 785‐nm laser, a photocurrent with equal magnitude but opposite polarity was generated at the left‐terminal graphene–Bi contact compared to that at the right terminal (middle panel of Figure S4b, Supporting Information). The photocurrents are nearly equal with maximum values but opposite signs at the two graphene–Bi contacts, approaching zero when the laser spot is focused at the middle of the channel (lower panel of Figure S4b, Supporting Information). This behavior persists when utilizing a 10.5‐µm laser as the excitation source (Figure S4c, Supporting Information). Thus, when the laser spot sizes of THz to MM waves surpass the device area, the symmetrical photocurrents at the two graphene–Bi contacts cancel each other out, resulting in a negligible net photocurrent.

The photocurrent characteristics of the flexible device were further measured at different illumination wavelengths with varying powers. As depicted in Figure 3d, the photocurrent demonstrates a clearly linear dependence on the laser power across different illumination wavelengths. The *R*_V_ of the photodetector can be calculated by:^[^ ^34^ ^]^

(3) $$R_{V}=\frac{V_{ph}}{P_{eff}}$$

where *V_ph_* is photovoltage and can be obtained by *V*_ph_ = *I*_ph_ × *R*_Gr_, with *I*_ph_ the photocurrent upon a specific illumination power, *R_Gr_* the resistance of the graphene channel. Parameter *P*_eff_ is the effective power, which is determined by the illumination power, laser spot size, and the area of the channel region (see Experimental Section for details on the calculation of *P*_eff_). The *R*_V_ increases against the illumination wavelength, which reaches 1.48 ± 0.02 mV W^−1^ at 550 nm in the visible (VIS) region and 9.0 ± 0.2 mV/W at 7.95 µm in the MIR region. Specifically, without the need for a THz antenna, the photodetector exhibits *R*_V_ of 33.7 ± 0.5 mV W^−1^ and 396.4 ± 5.1 mV W^−1^ at 119.0 µm (2.52 THz) and 1.11 mm (0.27 THz), respectively. The elimination of the need for a THz antenna is particular important for device miniaturization in the THz spectral regime, which can facilitate the development of highly integrated THz detector arrays on flexible substrates. The increase in the responsivity at longer wavelengths can be explained by considering that in the UV to NIR region, optical absorption in graphene is primarily dominated by interband transitions when *ħω* > 2*E_f_* (with *ω* the photon frequency). In contrast, intraband transitions take precedence in the infrared region due to the Pauli blocking effect at lower photon energies. At a constant power, longer‐wavelength radiation, characterized by a larger quantity of lower‐energy photons, can excite more electrons.^[^ ^36^ ^]^ Moreover, intraband transitions predominantly occur around the *E_f_*, while interband transitions involve energy bands that are far away from the *E_f_*. The higher density of electronic states in close proximity to the Fermi energy allows for the filling of more electrons (Figure S5, Supporting Information).^[^ ^37^ ^]^ Thus, the *R*_V_ increases with the illumination wavelength, attributed jointly to the contributions of both photon energies and the density of electronic states.

To further evaluate the photodetector's performance, we calculated its NEP and *D* ^*^. The NEP represents the minimum detectable power for a bandwidth of 1 Hz, with 1/*f* noise dominating the noise current at 1 Hz, as depicted in the noise spectrum (Figure S6, Supporting Information). Specifically, the NEP and *D* ^*^ can be calculated as,^[^ ^38^, ^39^ ^]^

(4) $$\mathrm{NEP}=\frac{i_{N}}{R_{I}}$$

(5) $$D*=\frac{\sqrt{A}\sqrt{B}}{\mathrm{NEP}}$$

where *i_N_* is noise current spectrum at 1 Hz bandwidth, *R_I_* is current responsivity, *A* is the effective area of the photodetector, and *B* is the bandwidth. The NEP (*D* ^*^) exhibits a decrease (increase) concerning the illumination wavelength (Figure 3f). Specifically, in the THz domain, in the absence of a THz antenna, the photodetector achieves an NEP of 100.7 ± 1.6 nW Hz^−^^0.5^ (8.6 ± 0.1 nW Hz^−^^0.5^) and a *D* ^*^ of 1459.6 ± 23.5 Jones (17 152.6 ± 220.5 Jones) at 119 µm (1.11 mm). These values are commendable in the context of THz photodetectors utilizing 2D materials.^[^ ^14^, ^15^, ^40^ ^]^ It should be noted that numerous graphene photodetectors demonstrate high *D* ^*^. Many of these devices leverage localized electromagnetic field enhancement effects from antennas, metallic nanostructures, or patterned graphene to bolster their *R*_V_ and *D* ^*^.^[^ ^14^, ^41^, ^42^ ^]^ However, in our flexible device, the monolayer graphene alone exhibits weak light absorption without these enhancements. Additionally, because the monolayer graphene is placed onto the flexible PET substrate, it is challenging to improve photodetection performance through post‐treatments such as thermal annealing. These factors contribute to a reduced *D* ^*^. Through optimization of fabrication processes and photodetector geometry, further enhancements in *R_V_*, *D* ^*^, and NEP can be achieved, as discussed below. In particular, by integrating the detector with additional metallic nanostructures and antennas, introducing the plasmonic effect of patterned graphene, and combining multiple devices into a photodetector array, the light absorption of the monolayer graphene can be augmented.^[^ ^43^ ^]^ As a result, much‐enhanced *R*_V_ and *D* ^*^ can be anticipated.

To assess the performance of the photodetector as a flexible device, the detector was subjected to different bending angle, where its photocurrent was characterized using an illumination wavelength of 119 µm (2.52 THz) (Figure 3h). Figure 3g illustrates the response of the photodetector at various bending angles (*θ*), where *θ* is defined as the angle between the tangents at the two terminals of the device after bending, as shown in the inset of Figure 3g. It is evident that the photodetector maintains a stable photocurrent as *θ* increases from 0° to 60°, allowing it to conformally adhere to surfaces with various bending profiles on the human body. Subsequently, we conducted the bending cycles test repeatedly over 1000 times at *θ* = 60° and captured the photocurrent at intervals of every 100 cycles to highlight its flexibility and repeatability (Figure S5, Supporting Information). Among these cycles, the typical photoresponse during bending cycles 950 to 1000 is presented in Figure 3g. Throughout the 1000 bending cycles, the photodetector maintains outstanding flexibility and stability, attributed to the inherent flexibility of the PET substrate and the excellent mechanical properties of graphene. The degradation of the photocurrent is only 3% after 1000 bending cycles, which can be attributed to the slight damage of the graphene–metal contact during the bending cycles. These results clearly highlight the potential of the photodetector for applications in flexible and wearable electronic devices.

**Figure 4:**
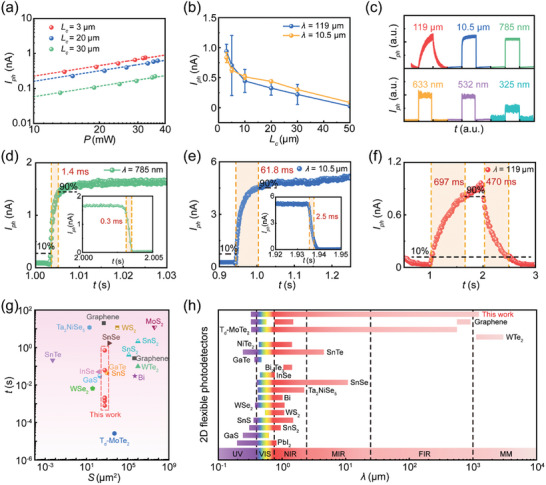
Flexible graphene photodetectors with different channel lengths. a) Dependence of the photocurrent on the illumination power at various channel lengths. Dots: experimental results. Lines: linear fittings. The illumination wavelength is *λ* = 119 µm. b) Dependence of the photocurrent on channel lengths. Dots: experimental results. Lines: guides for the eye. The illumination wavelengths are *λ* = 119 µm (blue line) and *λ* = 10.5 µm (yellow line). c) Time‐resolved photocurrent responses at different illumination wavelengths. d−f) Time‐resolved photocurrent responses at 785 nm (d), 10.5 µm (e), and 119 µm (f). g) Comparison of the response time and device area of the graphene flexible photodetector with flexible photodetectors based on other types of 2D materials. h) Comparison of the operation wavelength range of the graphene flexible photodetector with flexible photodetectors based on other types of 2D materials. The flexible photodetectors corresponding to (c−f) have a channel length of 30 µm.

In PTE process, a shorter diffusion length of hot carriers can enhance their collection. Therefore, by reducing the channel length, the *R*_V_ of the photodetector can be improved. **Figure** **4**a illustrates the dependence of the photocurrents, illuminated at the wavelength of 119 µm, on the excitation power measured from three graphene photodetectors with channel lengths of 3, 20, and 30 µm. A clear linear dependence is observed, and notably, the photocurrent increases as the channel length is reduced. Specifically, using 119‐µm (2.52‐THz) illumination, the photocurrent at *L*_c_ = 3 µm is 39 times greater than that observed at *L*_c_ = 50 µm (Figure 4b). Additionally, the *R_V_* and *D* ^*^ increase by factors of 548 and 1873, respectively, while the NEP decreases by a factor of 8689 as the channel narrows from 50 to 3 µm (Figure S8, Supporting Information). Most importantly, the ultra‐broadband spectral response is still maintained even in the smallest channel device (*L*_c_ = 3 µm, Figure S9, Supporting Information).

These performance enhancements are also closely tied to the pronounced localization of the electromagnetic field induced by the reduction in channel length. As the channels decrease in size, the two metal electrodes can concentrate the optical field to create hotspots that overlap with the photoactive region. This, in turn, significantly increases optical absorption and the generation of photocurrent. To quantitatively illustrate the relationship between the maximum field amplitude and channel length, finite‐difference time‐domain (FDTD) simulation was utilized to model electromagnetic field distributions across channel lengths ranging from 3 to 50 µm at wavelengths of *λ* = 119 µm and *λ* = 10.5 µm, respectively (Figure S10, Supporting Information). The simulation results reveal a substantial enhancement in electromagnetic field intensity as the channel length decreases. For instance, at *L*_c_ = 3 µm, the electromagnetic field intensity at *λ* = 119 µm increases by ≈8 times, leading to the generation of bright hotspots within the channel (inset of Figure S10, Supporting Information). These findings underscore the advantages of detector miniaturization, which can yield higher *R*_V_ and facilitate integration. However, as channel lengths shrink, the fabrication process becomes considerably challenging. The need for micro/nano fabrication equipment, such as electron beam lithography, increases both the cost and complexity of device fabrication, particularly for flexible substrates.

Another interesting issue refers to the differences in *R_V_*, NEP, and *D* ^*^ for illumination wavelengths of 10.5 and 119 µm as the channel length increases beyond 30 µm (Figure S8a–c, Supporting Information), where electromagnetic field enhancement effect is small for both wavelengths. Such differences in *R_V_*, NEP, and D* for illumination wavelengths of 10.5 and 119 µm can be attributed to contributions from both laser spot area and photon energy. Specifically, when the channel length exceeds 30 µm, the entire channel area is illuminated by the 119 µm‐wavelength laser (with a spot size ≈1 mm in diameter), while the spot of the 10.5 µm‐wavelength laser (≈21 µm in diameter) is smaller than the channel area. Upon 119‐µm illumination, both of the left and right graphene−electrode contacts are covered by the laser spot. The photocurrent generated at these two contacts partially cancels out. Additionally, under long‐wave (i.e., low photon energy) illumination, the excited electrons exhibit lower velocity than those excited by short‐wavelength illumination. As the channel becomes longer, the electrons experience multiple scattering, losing their energy and reducing the collected electrons. Consequently, electrons with lower velocity yield a smaller photocurrent compared to those with higher velocity, leading to a smaller number of collected electrons on average. Therefore, when the channel length exceeds 30 µm, the combined influence of a large laser spot area and low photon energy results in a lower *R_V_* at *λ* = 119 µm compared to that at *λ* = 10.5 µm.

Quantitatively, according to the definition of NEP and *D* ^*^ (Equations (4) and (5)), for a fixed channel length, they should be inverse and proportional to the *R_V_*, respectively. Therefore, for a specific channel length, the trends and numerical change proportions of *R_V_*, NEP, and *D* ^*^ should remain consistent under illumination with different wavelengths. In fact, when the channel length exceeds 20 µm, the influence of different wavelengths (10.5 and 119 µm) on *R_V_*, NEP, and *D* ^*^ is similar. To see this more clearly, we magnify the data region corresponding to channel length from 30 to 50 µm (Figure S8d–f, Supporting Information). Both the *R_V_* and *D* ^*^ at *λ* = 10.5 µm are 3.7 times those at *λ* = 119 µm, which is equal to the enhancement of NEP (inverse to the *R_V_*) at *λ* = 119 µm relative to that at *λ* = 10.5 µm.

The response time, a critical parameter characterizing the performance of the photodetector, was extracted from a single zero‐biased photoresponse curve directly captured by the high‐speed sampling oscilloscope at various wavelengths (Figure 4c and Experimental Section). The response time is defined as the duration between 10% and 90% of the rising (falling) edge of the photoresponse curve. At *λ* = 325 nm, the graphene flexible photodetector features a short response time of 0.8 ± 0.1 ms (rising edge) and 2.0 ± 0.1 ms (falling edge) (Figure S9a, Supporting Information), which is outstanding in current flexible photodetectors based on 2D materials (Table S1, Supporting Information). Such a fast response is benefit from the combination of high mobility in graphene and the PTE mechanism. Usually, the response time of graphene‐based PTE detectors is usually less than microseconds and the intrinsic speed of a graphene PTE photodetector can even reach nanoseconds or picoseconds.^[^ ^32^ ^]^ The millisecond response time of our graphene photodetector can be ascribed to the light absorption from the flexible substrate. Previously, graphene PTE detectors are commonly fabricated on silicon substrate which typically has a low absorption for incident light,^[^ ^32^, ^34^, ^44^ ^]^ thereby exerting minimal influence on the response speed of graphene detectors. In our device fabricated onto the PET substrate, the light absorption of PET would result in a uniform heating of the carriers in the channel, which amplifies the scattering rate of the carriers and thus violates the carrier diffusion to the collection electrode. For the same reason, for most flexible 2D detectors, the response time commonly falls within the range of milliseconds to seconds (Table S1, Supporting Information). It is noted that the response times are found to increase against the wavelengths, which are 2.0 ± 0.2 ms, 2.1 ± 0.2 ms, 1.4 ± 0.1 ms, 61.8 ± 1.5 ms, and 697 ± 13 ms at illumination wavelengths of 532 nm, 633 nm, 785 nm, 10.5 µm, and 119 µm, respectively (Figure 4d−f; Figure S11b,c, Supporting Information). This behavior can also be ascribed to the increased absorption of the flexible PET substrate in the MIR to THz region.^[^ ^45^ ^−^ ^47^ ^]^ The heated carriers in the channel due to the PET absorption will suppress the carrier temperature gradient and, correspondingly, extends the diffusion time. Nevertheless, our device maintains a favorable position among currently reported 2D flexible detectors in terms of size and response time (Figure 4g; Table S1, Supporting Information).^[^ ^6^, ^17^ ^−^ ^20^, ^27^, ^48^ ^−^ ^58^ ^]^ Notably, the detectable wavelength range of this device, spanning the UV to MM wave spectrum, is the broadest among all 2D flexible detectors reported to date (Figure 4h; Table S1, Supporting Information).^[^ ^6^, ^16^, ^18^ ^−^ ^20^, ^26^, ^27^, ^48^, ^50^ ^−^ ^55^, ^58^ ^−^ ^62^ ^]^ This strongly underscores its potential applications in the development of broadband and miniaturized wearable photodetectors with fast response.

## 5 Ultra‐Broadband Imaging of the Flexible Photodetectors Under Simulated Human Body‐Wearing Conditions

**Figure 5:**
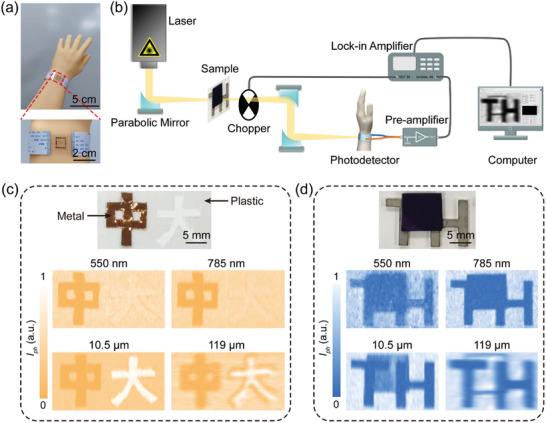
Broadband imaging of the flexible graphene photodetector under simulated human body‐wearing conditions. a) Photograph of the flexible graphene photodetector affixed to a human wrist model. b) Schematic showing the dual‐focus scanning imaging system used for broadband imaging of objects. c) Photocurrent imaging of two Chinese characters, “中” and “大” using various illumination wavelengths. The two characters are formed from metal and hollowed out on a plastic substrate, respectively. d) Photocurrent imaging of a “TH”‐shaped metal plate hidden behind an opaque high‐resistance silicon slab using various illumination wavelengths.

The broadband optical imaging capabilities of a photodetectors that are able to conform to surfaces with random curvature play a vital role in the development of future wearable optoelectronic devices. To validate the practical applications of the flexible graphene photodetector, we conducted ultra‐broadband imaging under illumination at 550 nm, 785 nm, 10.5 µm, and 119 µm, respectively, utilizing the flexible photodetector affixed to a human wrist model (**Figure** **5**a). For this purpose, a dual‐focus scanning imaging system was employed. As illustrated in Figure 5b, the objects were mounted onto a 2D electronically controlled displacement platform positioned at the focal plane of the first parabolic mirror. Simultaneously, the photodetector, affixed to the human wrist model, was placed at the focus of the third parabolic mirror. By raster scanning the object in the focal plane and collecting the photocurrent generated by illuminating the photodetector with transmitted light through the object, a 2D image of the object could be reconstructed (see Experimental Section for details).

Figure 5c depicts photocurrent images of two Chinese characters, “中” (meaning “middle”) and “大” (meaning “large”), collected at different illumination wavelengths. These characters were respectively formed from metal and hollowed out on a plastic substrate (Figure 5c, upper panel). The images consist of pixels measuring 90 × 50, with each pixel corresponding to a step size of 0.3 mm × 0.3 mm. Under VIS (550 nm) and NIR (785 nm) illuminations, the metal character blocks the incident light while the hollowed character remains transparent. Because the plastic substrate has negligible absorption at these two wavelengths, only the character “中” can be resolved (Figure 5c, middle panel). In contrast, under 10.5 and 119‐µm illumination, the continues to block the incident light, while the plastic absorbs a portion of the incident light, leading to noticeable contrast for both characters “中” and “大” (Figure 5c, lower panel). Importantly, due to the different absorption characteristics of metal, plastic, and air in response to MIR and THz waves, it is possible to distinguish between the two characters based on the image contrast. This result clearly highlights the capability of the photodetector in discriminating substances.

The MIR and THz photoresponses of the graphene flexible photodetector make it possible to identify shielded objects, demonstrating potential applications in security inspection and industrial non‐invasive testing. As a proof of concept, we utilized the flexible photodetector to image a “TH”‐shaped metal plate hidden behind an opaque high‐resistance silicon slab (Figure 5d, upper panel). Photocurrent images at different illumination wavelengths were obtained with 70 × 50 pixels, clearly outlining the metal and silicon slab (Figure 5d, middle and lower panels). Compared to images obtained at 550 nm and 785 nm, the hidden “TH” pattern becomes visible in images obtained at 10.5 and 119 µm. Particularly, in the image obtained at 119 µm, the silicon slab appears nearly transparent or as if it is not present. This is attributed to the THz wave easily penetrating through the high‐resistance silicon with negligible absorption. The results presented in Figure 5c,d clearly indicate that, owing to the ultra‐broadband photoresponse of the flexible graphene detector, it can not only distinguish different materials but also identify concealed objects based on their shapes and varied optical absorption across a broad spectral range.

An important issue is that for a practical human body application, the temperature of the human wrist may heat the device and affect its performance, for example, reducing the speed and *R*_V_ due to suppression of the temperature gradient along the channel region. To address this issue, the flexible device can be further optimized in practical applications by integrating the heat dissipation system or improving its *R*_V_. First, a suitable flexible layer can be added between the device and the human body to isolate the device from the human heat source and reduce thermal conduction. Second, the flexible device can be integrated with temperature feedback sensors and thermal modulation devices to manage the temperature rise. Thirdly, the *D* ^*^ can be further improved through the integration of metallic nanostructures, antennas, patterned graphene, or multiple such devices into a photodetector array.

## 6 Conclusion

In summary, we have successfully developed a flexible and wearable graphene photodetector utilizing the hot‐carrier PTE effect induced by dissimilar metal electrodes. This self‐powered photodetector demonstrates excellent performance, featuring a rapid response time of 0.8 ± 0.1 ms and an ultra‐broadband photoresponse covering a wide spectrum from UV to MM wavelengths (325 nm to 1.19 mm). It is noteworthy that it possesses the broadest response spectrum among all reported 2D flexible photodetectors to date, and simultaneously, it boasts one of the fastest response speeds among these devices. By leveraging the electromagnetic confinement provided by the electrodes, the photodetector's *R*_V_ can be enhanced through the reduction of the channel length. Under simulated human body‐wearing conditions, our flexible photodetector enables wide‐spectrum imaging of distinct and shielded objects at wavelengths of 550 nm, 785 nm, 10.5 µm, and 119 µm. Looking ahead, the integration of multiple such devices into a photodetector array holds the potential to enlarge the imaging photosensitive area and sensitivity. This could lead to comprehensive imaging capabilities around the human body. The advantage of omnidirectional imaging, achieved without the need for complex and bulky systems, positions our approach favorably compared to traditional rigid detectors. We anticipate that these results will significantly contribute to the development of miniaturized and wearable optoelectronic devices spanning the UV to THz region.

## 7 Experimental Section

### Graphene Transfer and Device Fabrication

The PET substrate, known for its high transparency and resistance to organic solvents, exhibits excellent stability, flexibility, and insulation properties as a flexible substrate.^[^ ^63^ ^]^ A monolayer graphene film (Jiangsu XFNANO Materials Tech. Co., Ltd.), coated with polymethyl methacrylate (PMMA) as a mask, was transferred onto the PET substrate using a wet transfer technique. Subsequently, it underwent a 1‐hour immersion in acetone to remove the PMMA. The monolayer graphene was patterned using a UV photolithography technique (TuoTuo Technology) and then etched by oxygen plasma (O_2_ at 400 Pa, power of 18 W, etching for 2 min) to create a graphene channel. Following this, one of the electrodes was defined using UV photolithography and coated with Bi/Au (ZhongNuo Advanced Material (Beijing) Technology Co., Ltd.) with thicknesses of 10 nm/100 nm through thermal evaporation. After the lift‐off process, the second electrode underwent a series of procedures involving UV photolithography and subsequent deposition of Cr/Au (10 nm/100 nm) (details in Figure S1, Supporting Information). The flexible graphene photodetector, featuring asymmetric electrodes, was then affixed to a flexible printed circuit board (PCB). The two electrodes were connected to the PCB pads by bonding aluminum wires using silver paste for further photoresponse characterizations.

### Characterizations

The graphene's morphology and Raman spectrum were characterized using a confocal Raman spectrometer (Renishaw, Invia Reflex). The electrical transport characteristics of the photodetector were measured using a sourcemeter (Tektronix, Keithley 2636b). Various light sources spanning from UV to NIR regions were employed, including a 325‐nm helium‐cadmium laser (Renishaw), a 532‐nm solid‐state laser (Renishaw), a 633‐nm helium‐neon laser (Renishaw), a 785‐nm semiconductor laser (Leooptics, LE‐LS‐785‐90TSMF), and a supercontinuum white‐light laser (Fianium, SC400‐4‐PP) integrated with different narrow‐band filters. Additionally, tunable MIR quantum cascade lasers (Block Engineering, Q0295) and a 10.5‐µm MIR quantum cascade laser (Tuotuo Technology, TTT‐00‐MIR) were utilized for characterizing photocurrent generation at MIR spectral region. For THz source, a far‐infrared gas laser (Edinburgh Instruments, FIRL 100) with output frequencies of 3.11 THz (96 µm), 2.52 THz (119 µm), and 1.84 THz (163 µm) was employed. Sub‐MM to MM wave photodetection measurement was performed using a microwave signal source (Rohde & Schwarz, SMB 100A) integrated with a frequency multiplier (VDI, SGX) and a tripler, covering central frequencies ranging from 0.35 THz (857 µm) to 0.25 THz (1.19 mm). The modulation frequency of the lasers was set by an optical chopper (SCITEC, 310CD). To record the photoresponses of the detector, a low‐noise current pre‐amplifier (FEMTO, DLPCA‐200) was used to amplify the signals, which were then read out by a lock‐in amplifier (Stanford, SR830) and a digital phosphor oscilloscope (Tektronix, DPO7354C). The noise current spectrum of the photodetector was measured using a semiconductor parameter analyzer (PDA FS‐Pro).

### Photoresponse Measurements

In the time‐resolved photoresponse measurement, a laser beam, modulated by a shutter with a 2‐second on‐off switching period, was precisely focused at the graphene–Bi contact. Fine adjustment was performed to maximize the photocurrent. The photocurrent generated was then converted into a voltage signal using a current pre‐amplifier and recorded by a digital phosphor oscilloscope operating at a sampling frequency of 2.5 GHz. For the SPCM measurement, a laser beam, modulated by an optical chopper at 24 Hz, was focused onto the device through an optical microscope with a 40× objective lens (N.A. = 0.5). The device was scanned using a 2D electrically controlled displacement stage with a step of 1 µm × 1 µm. The photocurrents at the corresponding positions were measured by a current pre‐amplifier and recorded by a lock‐in amplifier. The resulting photocurrent signals were plotted to generate images depicting the photocurrent distribution across the device. In the 2D imaging of a specific object, the transmitted light from the imaging object, illuminated by a laser beam modulated at 24 Hz, was directed onto the device to generate photocurrent. The object was scanned using a 2D electrically controlled displacement stage with a step size of 0.3 mm × 0.3 mm. The photocurrents corresponding to the specific position of the object were measured by a current pre‐amplifier and recorded by a lock‐in amplifier. By mapping the distribution of photocurrent with scanning positions, a 2D image of the object could be obtained.

### Simulation

The electromagnetic field distribution of the electrodes at 119 µm (2.52 THz) and 10.5 µm were simulated using FDTD. The electrodes were irradiated with a perpendicular incident plane wave of linear polarization. In the simulation, the dielectric function of the PET substrate was adopted from literature.^[^ ^64^ ^]^ The Au electrode exhibited a high electrical conductivity in MIR and THz region, which can be considered as a perfect electric conductor (PEC).^[^ ^65^, ^66^ ^]^

### Responsivity Calculation

The *R*_V_ can be calculated by:

(6) $$R_{V}=\frac{V_{ph}}{P_{eff}}=\frac{I_{ph}\times R_{Gr}}{P_{eff}}$$

where *R*_Gr_ is the resistance of the graphene channel, *P*_eff_ is the effective power, *V*_ph_ and *I*_ph_ are the actual photovoltage and photocurrent, respectively. In the experiment, the actual photocurrent can be obtained by:^[^ ^14^ ^]^

(7) $$I_{ph}=\frac{2\pi \sqrt{2}V_{lock}}{4G}$$

where *V*_lock_ is the photovoltage read out by the lock‐in amplifier, and *G* is the gain of the current preamplifier in V/A. In the UV to MIR region, the laser spot area is smaller than the device channel area, so the effective power can be expressed by:

(8) $$P_{eff}=P_{0}$$

where *P*_0_ is the total incident power. In the far‐infrared to MM region, the laser spot area is larger than the device channel area, so the effective power can be calculated by:

(9) $$P_{eff}=P_{0}\times \frac{S_{device}}{S_{0}}$$

where *S*_device_ is the effective absorption area of the photodetector, and *S*_0_ is the laser spot area. For the photodetector, *S*_device_ was defined as the graphene channel area.

### Statistical Analysis

Raman mapping and photocurrent imaging were normalized in Raman intensity and photocurrent intensity, respectively. The fitting of the photocurrent and power was performed using the linear model. All statistical analysis and data processing were conducted using Excel 2023 software. Results were represented as means ± SD or as specified.

## Conflict of Interest

The authors declare no conflict of interest.

## Supporting information

Supporting Information

## Data Availability

The data that support the findings of this study are available from the corresponding author upon reasonable request.
