# Supplementary material for: A Flexible and Wearable Photodetector Enabling Ultra‐Broadband Imaging from Ultraviolet to Millimeter‐Wave Regimes
Source: Adv Sci (Weinh). 2024 Apr 24;11(26):2401631. doi: 10.1002/advs.202401631 (PMC11234453; doi:10.1002/advs.202401631)
Supplement: Supplementary file 1 — Supporting Information [file ADVS-11-2401631-s001.pdf]

ADVANCED SCIENCE
Open Access

Supporting Information

for *Adv. Sci.*, DOI 10.1002/advs.202401631

A Flexible and Wearable Photodetector Enabling Ultra-Broadband Imaging from Ultraviolet to Millimeter-Wave Regimes

Shaojing Liu, Ximiao Wang, Ningsheng Xu, Runli Li, Hai Ou, Shangdong Li, Yongsheng Zhu, Yanlin Ke, Runze Zhan, Huanjun Chen and Shaozhi Deng*

Supporting Information

A Flexible and Wearable Photodetector Enabling Ultra-Broadband Imaging from Ultraviolet to Millimeter-Wave Regimes

Shaojing Liu, Ximiao Wang, Ningsheng Xu, Runli Li, Hai Ou, Shangdong Li, Yongsheng Zhu, Yanlin Ke, Runze Zhan, Huanjun Chen, and Shaozhi Deng*

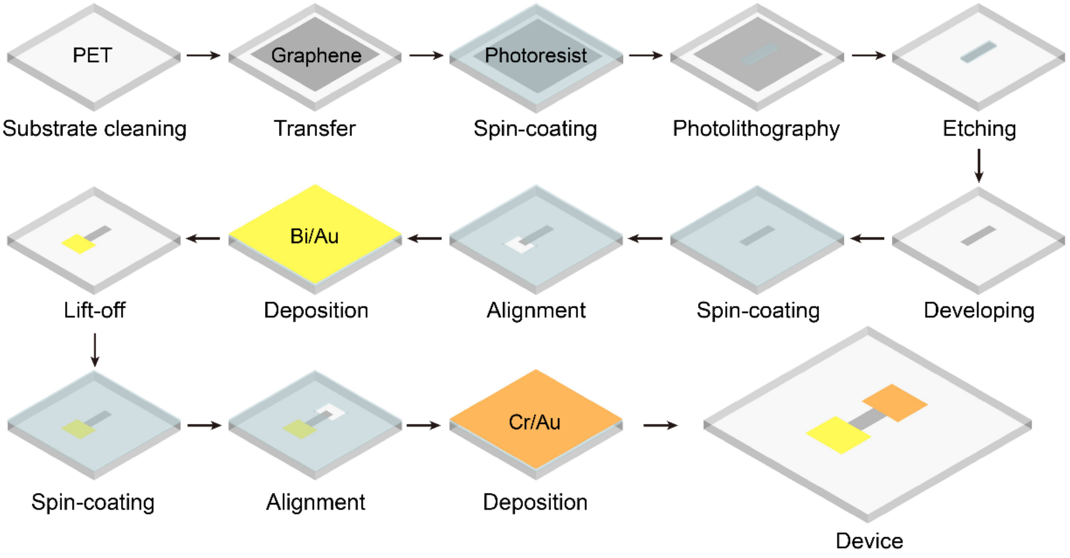

Figure S1. Schematic showing the fabrication processes of the flexible graphene photodetector.

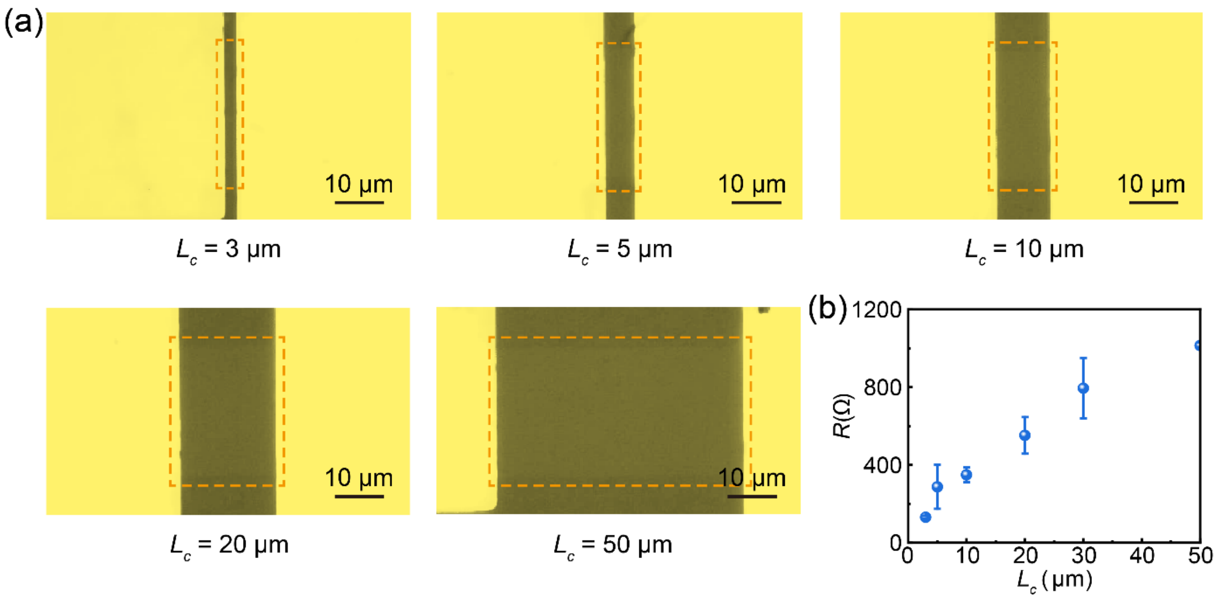

Figure S2. Electrical characterization of the flexible graphene photodetectors with different channel lengths (L_c). a) Optical microscope images of the flexible graphene photodetectors with L_c of 3 μm, 5 μm, 10 μm, 20 μm, and 50 μm. b) Resistances (R) of flexible photodetectors with varied L_c. For each channel length, error bars are statistics from 4 similar devices.

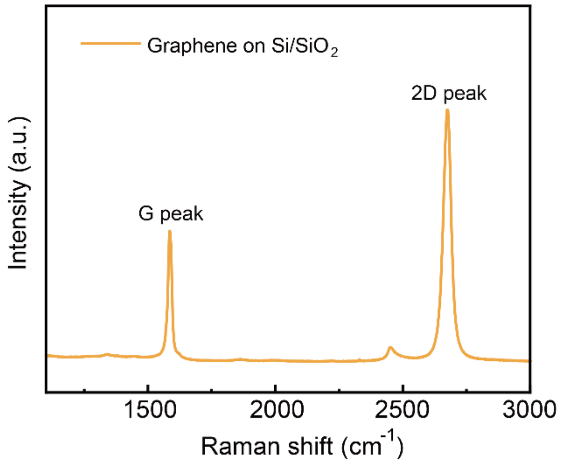

Figure S3. Raman spectrum of a monolayer graphene on Si/SiO_2 substrate.

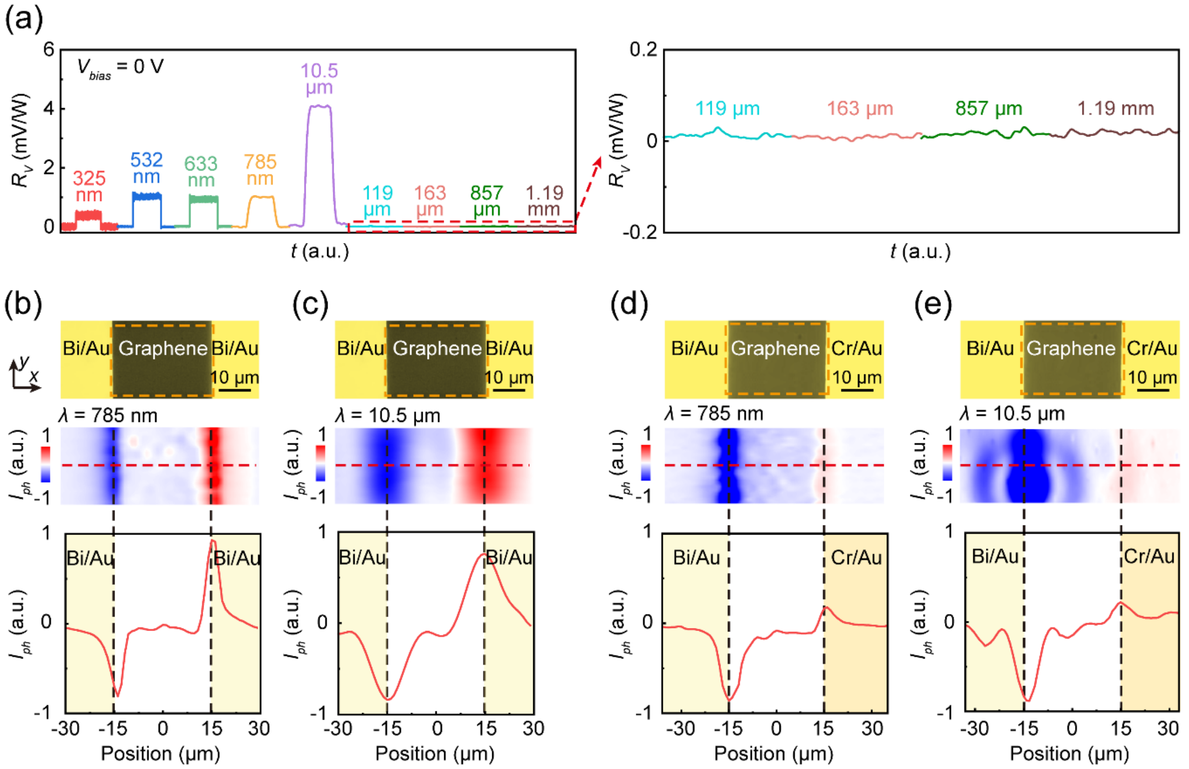

Figure S4. Photocurrent of the flexible graphene photodetectors with symmetric and asymmetric electrodes. a) Left: time-resolved photocurrent responses of the Bi/Au–graphene–Bi/Au photodetector in a broadband spectrum. Right: the magnified views of the regions enclosed by red dashed boxes shown in the left panel. b–e) Optical microscope image (upper panel), scanning photocurrent microscopy image (middle panel), and position-dependent photocurrent profile (lower panel) along the midline of the Bi/Au–graphene–Bi/Au

photodetector (b, c) and Bi/Au–graphene–Cr/Au photodetector (d, e). The photocurrent images in (b, d) and (c, e) were collected using illumination wavelengths of 785 nm and 10.5 μm without bias, respectively. The black and red dashed lines mark the graphene–metal contacts and the midline of the photodetector, respectively.

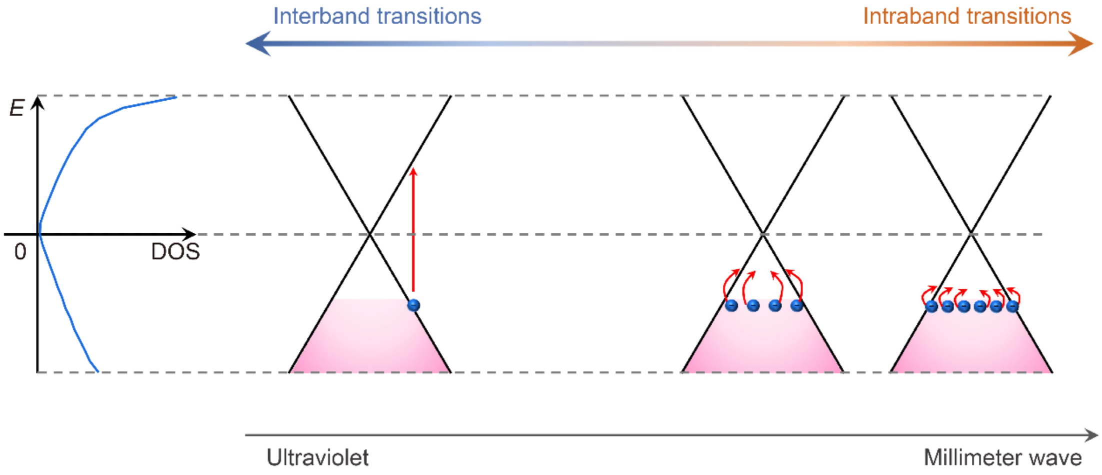

Figure S5. Left: schematic showing the density of states (DOS) varies with energy (*E*). Right: schematic representing electronic interband and intraband transitions in graphene upon illumination with wavelengths spanning ultraviolet (UV) to millimeter (MM) wave regions.

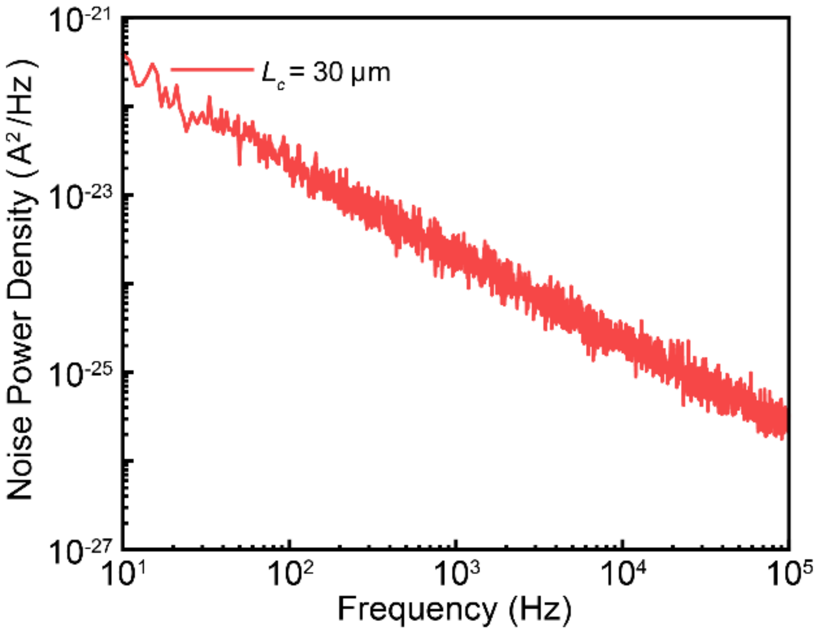

Figure S6. Current noise power density of the flexible graphene photodetector.

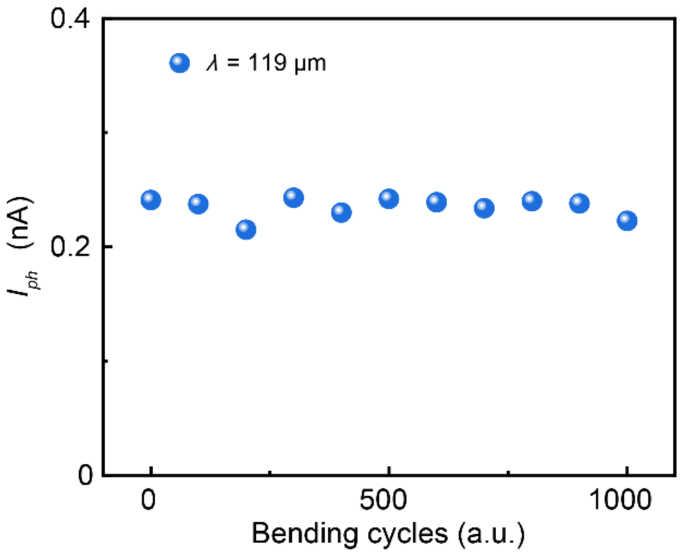

Figure S7. Photocurrent (I_{ph}) of the flexible graphene photodetector at λ = 119 µm during bending cycles of 1000 times. The bending angle is 60°.

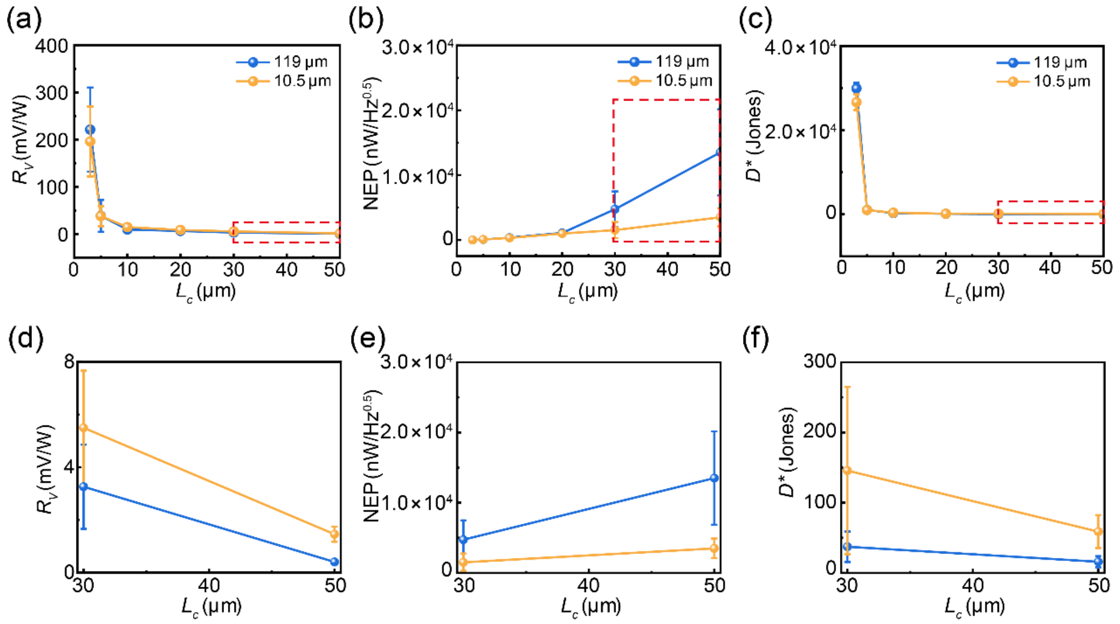

Figure S8. Photodetection performances of the flexible graphene photodetector with different L_c. a) Responsivity (R_V). b) Noise equivalent power (NEP). c) Specific detectivity (D^*). d–f) The magnified views of the regions enclosed by red dashed boxes shown in (a–c). The illumination wavelengths are λ = 119 µm (blue lines) and λ = 10.5 µm (orange lines).

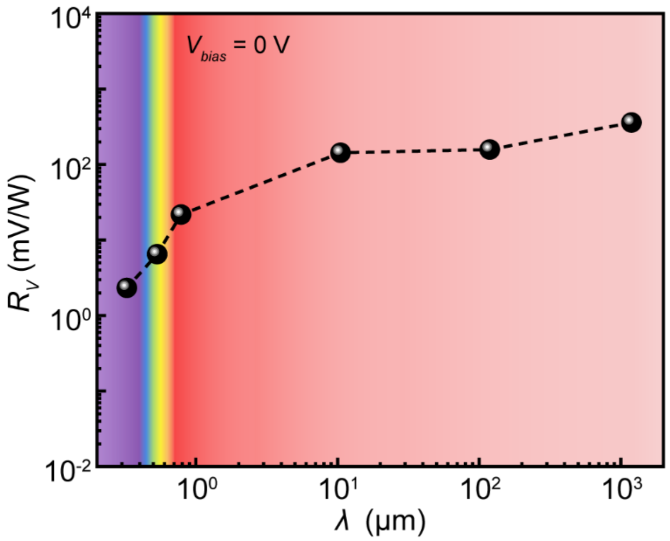

Figure S9. Wavelength-dependent responsivity of the flexible graphene photodetector with L_c = 3 μm.

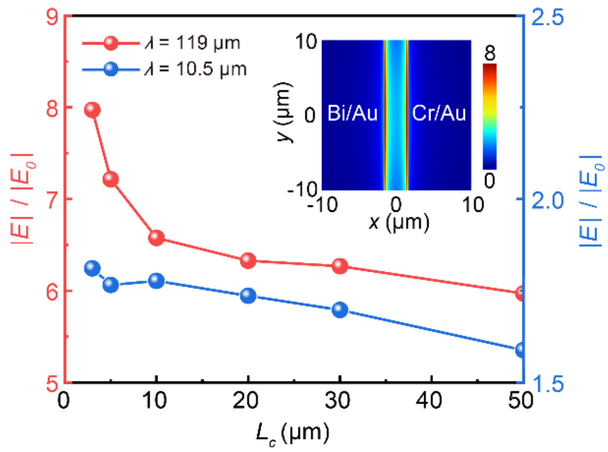

Figure S10. Simulated electromagnetic field enhancements at different L_c. The localized field enhancement, $|E|/|E_0|$, is recorded at the position near the electrode–graphene contact, where $|E|$ is maximum. The illumination wavelengths are λ = 119 μm (red dots) and λ = 10.5 μm (blue dots). Inset: typical electromagnetic field distribution at λ = 119 μm in the channel region, where the channel length is 3 μm.

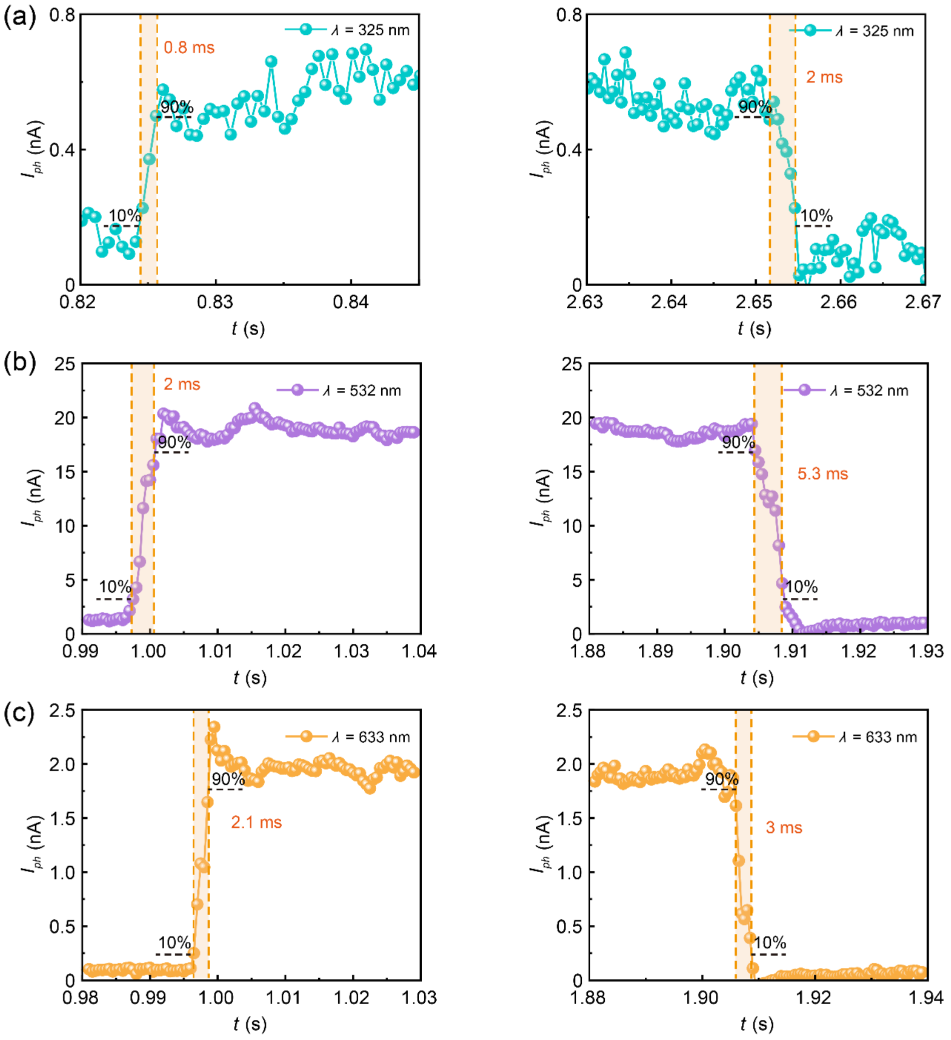

Figure S11. Response time (t) of the flexible graphene photodetector at different illumination wavelengths.

Table S1. Comparison of performances of flexible photodetectors based on 2D materials.

Materials	R_V / R_I	λ	t (s)	S (μm^2)	Thickness (nm)	D^* (Jones)	Imaging	Bending testing
Graphene (this work)	0.4 V/W	325 nm – 1.19 mm	8×10^{-4}	600	0.34	1.7×10^4	Yes	1000 cycles
Graphene [1]	0.11 A/W	405 nm – 635 nm	0.269	4.2×10^5	100 – 500	-	No	-
Graphene [2]	2 V/W	600 μm – 909 μm	-	5900	0.34	-	No	-

Graphene [3]	556 A/W	320 nm – 540 nm	-	-	-	6.0 × 10^{11}	No	25000 cycles
Graphene [4]	-	IR lamp (> 780 nm)	20.1	480	6	-	No	
BP [5]	53 A/W	830 nm	-	2	10	-	No	1000 cycle
T_d-$MoTe_2$ [6]	5.3 × 10^{-4} A/W	325 nm – 566 µm	2.6 × 10^{-5}	5250	4	-	Yes	-
WTe_2 [7]	1.54×10^8 V/W	1.07 mm – 3 mm	0.098	1.0 × 10^6	320	-	No	500 cycles
$NiTe_2$ [8]	13.27 A/W	450 nm – 1550 nm	0.31	-	38 – 90	2.2 × 10^{11}	No	20 cycles
SnTe [9]	71.11 A/W	254 nm – 4650 nm	0.21	5.25 × 10^{-3}	> 120	-	No	80 cycles
GaTe [10]	0.03 A/W	375 nm – 473 nm	0.054	740	83	-	No	200 cycles
Bi_2Te_3 [11]	58 A/W	1064 nm – 1550 nm	0.11	-	10	6.1 × 10^8	No	-
InSe [12]	12.3 A/W	450 nm – 785 nm	0.05	150	12	5.47 × 10^{10}	No	-
SnSe [13]	0.16 A/W	404 nm – 10.6 µm	1.7	1600	15	3.9 × 10^7	No	200 cycles

In_2Se_3 [14]	1650 A/W	633 nm	-	40	-		No	360 cycles
Ta_2NiSe_5 [15]	280 A/W	405 nm – 2200 nm	11.7	20	18	3.8×10^9	No	1000 cycles
Bi [16]	4.8×10^{-3} A/W	405 nm – 1064 nm	0.03	5.0×10^5	13	-	No	100 cycles
WSe_2 [17]	0.92 A/W	370 nm – 1064 nm	0.9	-	48	-	No	-
WSe_2 [18]	2.46 A/W	405 nm – 980 nm	6.51×10^{-3}	40	22	7.6×10^{10}	No	500 cycles
GaSe [19]	0.03 A/W	White light	-	40	0.8	-	No	
WS_2 [20]	4.04×10^{-3} A/W	532 nm – 1064 nm	11.6	1.0×10^4	< 100	2.55×10^9	No	200 cycles
SnS [21]	1.28×10^3 A/W	355 nm – 1550 nm	0.044	1000	26	3.02×10^{11}	No	-
SnS_2 [22]	2.06×10^{-4} A/W	300 nm-830 nm	0.4	1.3×10^5	15	-	No	
SnS_2 [23]	0.0408 A/W	405 nm – 980 nm	2.1	1×10^6	280	-	No	4000 cycles
MoS_2 [24]	0.02 A/W	532 nm	12	4×10^7	1	-	No	-
hBN [25]	5.2×10^{-5} A/W	185 nm	0.267	-	2	8.05×10^{10}	No	200 cycles
GaS [26]	19.2 A/W	254 nm –	0.03	200	0.8 – 5	10^{13} – 10^{14}	No	20 cycles

		610 nm						
PbI_2 [27]	147.6 A/W	200 nm – 800 nm	0.018	-	8.32	2.56 × 10^{11}	No	100 cycles
MoS_2-Graphene [28]	4.5 A/W	515 nm – 850 nm	-	~ 4.0 × 10^4	6	-	Yes	-
PbI_2-Graphene [29]	45 A/W	480 nm	3.5 × 10^{-5}	-	95	-	Yes	100 cycles
Bi_2Te_3-SnS-Bi_2Te_3 [30]	115 A/W	370 nm – 808 nm	-	1.75 × 10^9	30	4.1 × 10^{11}	No	100 cycles

References

[1] P. Kang, M.C. Wang, P.M. Knapp, S. Nam, *Adv. Mater.* **2016**, *28*, 4639.

[2] X. Yang, A. Vorobiev, A. Generalov, M.A. Andersson, J. Stake, *Appl. Phys. Lett.* **2017**, *111*, 021102.

[3] Q.-M. Wang, Z.-Y. Yang, *Carbon* **2018**, *138*, 90.

[4] N. Liu, H. Tian, G. Schwartz, J.B.H. Tok, T.-L. Ren, Z. Bao, *Nano Lett.* **2014**, *14*, 3702.

[5] J. Miao, B. Song, Q. Li, L. Cai, S. Zhang, W. Hu, L. Dong, C. Wang, *ACS Nano* **2017**, *11*, 6048.

[6] Q. Yang, X. Wang, Z. He, Y. Chen, S. Li, H. Chen, S. Wu, *Adv. Sci.* **2023**, *10*, e2205609.

[7] Q. Song, Y. Zhou, Y. Wang, F. Gao, J. Wang, M. Zhang, Y. Wang, B. Zhang, P. Yan, B. Dong, *Adv. Electron. Mater.* **2023**, *9*, 2300149.

[8] Y. Zhang, L. Chen, T. Ding, J. Xu, X. Zhang, H. Liu, G. Chen, *ACS Appl. Nano Mater.* **2022**, *5*, 6094.

[9] J. Yang, W. Yu, Z. Pan, Q. Yu, Q. Yin, L. Guo, Y. Zhao, T. Sun, Q. Bao, K. Zhang, *Small* **2018**, *14*, 1802598.

[10] Z. Wang, M. Safdar, M. Mirza, K. Xu, Q. Wang, Y. Huang, F. Wang, X. Zhan, J. He, *Nanoscale* **2015**, *7*, 7252.

[11] A. Pandey, R. Yadav, M. Kaur, P. Singh, A. Gupta, S. Husale, *Sci. Rep-UK* **2021**, *11*, 832.

[12] S.R. Tamalampudi, Y.-Y. Lu, R.K. U, R. Sankar, C.-D. Liao, K.M. B, C.-H. Cheng, F.C. Chou, Y.-T. Chen, *Nano Lett.* **2014**, *14*, 2800.

[13] H. Xu, L. Hao, H. Liu, S. Dong, Y. Wu, Y. Liu, B. Cao, Z. Wang, C. Ling, S. Li, Z. Xu, Q. Xue, K. Yan, *ACS Appl. Mater. Interfaces* **2020**, *12*, 35250.

[14] W. Zheng, T. Xie, Y. Zhou, Y.L. Chen, W. Jiang, S. Zhao, J. Wu, Y. Jing, Y. Wu, G. Chen, Y. Guo, J. Yin, S. Huang, H.Q. Xu, Z. Liu, H. Peng, *Nat. Commun.* **2015**, *6*, 6972.

[15] T. Guo, Z. Sa, P. Wei, Y. Jian, X. Chen, Z. Chen, J. Avila, P. Dudin, Z.-x. Yang, X. Song, F. Liu, S. Zhang, *2D Mater.* **2023**, *10*, 025004.

[16] Q. Zhou, D. Lu, H. Tang, S. Luo, Z. Li, H. Li, X. Qi, J. Zhong, *ACS Appl. Electron. Mater.* **2020**, *2*, 1254.

[17] Z. Zheng, T. Zhang, J. Yao, Y. Zhang, J. Xu, G. Yang, *Nanotechnology* **2016**, *27*, 225501.

[18] Y. Ma, D. Liu, J. Hao, L. Wang, W. Wang, *AIP Adv.* **2020**, *10*, 125027.

[19] Y. Zhou, Y. Nie, Y. Liu, K. Yan, J. Hong, C. Jin, Y. Zhou, J. Yin, Z. Liu, H. Peng, *ACS Nano* **2014**, *8*, 1485.

[20] J. Li, J. Han, H. Li, X. Fan, K. Huang, *Mat. Sci. Semicon. Proc.* **2020**, *107*, 104804.

[21] A. Dong, T. Tian, H. Zhao, S. Li, S. Deng, X. Song, Y. Yan, C. Xia, J. Li, *Semicond. Sci. Tech.* **2020**, *35*, 115016.

[22] Y. Tao, X. Wu, W. Wang, J. Wang, *J. Mater. Chem. C* **2015**, *3*, 1347.

[23] Y. Lei, J. Luo, X. Yang, T. Cai, R. Qi, L. Gu, Z. Zheng, *ACS Appl. Mater. Interfaces* **2020**, *12*, 24940.

[24] F. Yu, M. Hu, F. Kang, R. Lv, *Prog. Nat. Sci.* **2018**, *28*, 563.

[25] C.-H. Lin, H.-C. Fu, B. Cheng, M.-L. Tsai, W. Luo, L. Zhou, S.-H. Jang, L. Hu, J.-H. He, *npj 2D Mater. Appl.* **2018**, *2*, 23.

[26] P. Hu, L. Wang, M. Yoon, J. Zhang, W. Feng, X. Wang, Z. Wen, J.C. Idrobo, Y. Miyamoto, D.B. Geohegan, K. Xiao, *Nano Lett.* **2013**, *13*, 1649.

[27] M. Zhong, L. Huang, H.-X. Deng, X. Wang, B. Li, Z. Wei, J. Li, *J. Mater. Chem. C* **2016**, *4*, 6492.

[28] C. Choi, M.K. Choi, S. Liu, M.S. Kim, O.K. Park, C. Im, J. Kim, X. Qin, G.J. Lee, K.W. Cho, M. Kim, E. Joh, J. Lee, D. Son, S.H. Kwon, N.L. Jeon, Y.M. Song, N. Lu, D.H. Kim, *Nat. Commun.* **2017**, *8*, 1664.

[29] J. Zhang, Y. Huang, Z. Tan, T. Li, Y. Zhang, K. Jia, L. Lin, L. Sun, X. Chen, Z. Li, C. Tan, J. Zhang, L. Zheng, Y. Wu, B. Deng, Z. Chen, Z. Liu, H. Peng, *Adv. Mater.* **2018**, *30*, 1803194.

[30] J. Yao, G. Yang, *Small* **2018**, *14*, 1704524.
